# Capsaicin protects cardiomyocytes against lipopolysaccharide-induced damage via 14-3-3γ-mediated autophagy augmentation

**DOI:** 10.3389/fphar.2021.659015

**Published:** 2021-04-27

**Authors:** Yang Qiao, Liang Wang, Tianhong Hu, Dong Yin, Huan He, Ming He

**Affiliations:** ^1^ Institute of Cardiovascular Diseases, Jiangxi Academy of Clinical Medical Sciences, The First Affiliated Hospital of Nanchang University, Nanchang, China; ^2^ Department of Rehabilitation, The First Affiliated Hospital of Nanchang University, Nanchang, China; ^3^ Jiangxi Provincial Key Laboratory of Basic Pharmacology, Nanchang University School of Pharmaceutical Science, Nanchang, China; ^4^ Jiangxi Provincial Key Laboratory of Molecular Medicine, The Second Affiliated Hospital, Nanchang University, Nanchang, China

**Keywords:** 14-3-3γ, Autophagy, Cardiac dysfunction, Capsaicin, lipopolysaccharide, Mitochondria

## Abstract

**Background:** The myocardium is susceptible to lipopolysaccharide (LPS)-induced damage in sepsis, and cardiac dysfunction is a leading cause of mortality in patients with sepsis. The changes in cardiomyocyte autophagy in sepsis and the effects and mechanism of action of capsaicin (Cap) remain unclear.

**Methods and Results:** The potential pathway of 14-3-3γ-dependent autophagy and the effects and mechanisms of Cap were studied in LPS-induced injury to primary cultured neonatal rat cardiomyocytes. The results showed that cardiomyocyte viability decreased, lactate dehydrogenase and creatine kinase activities increased, 14-3-3γ expression was downregulated, and autophagy was inhibited after LPS challenge. Cap pretreatment augmented autophagy by upregulating 14-3-3γ expression and activating AMP-activated protein kinase (AMPK) and unc-51 like autophagy-activating kinase 1 (ULK1), suppressing mammalian target of rapamycin (mTOR), alleviating cardiac dysfunction and improving the inflammation response, whereas pAD/14-3-3γ-shRNA nullified the above effects. Cap pretreatment also decreased the levels of IL-1β, TNF-α, IL-6, and IL-10; suppressed intracellular oxidative stress; reduced the intracellular/mitochondrial reactive oxygen species (ROS); balanced GSH/GSSG; increased GSH-Px, catalase, and SOD activities; and decreased MDA contents. It also increased ATP content, activated complex Ⅰ and complex Ⅲ, stabilized the mitochondrial membrane potential, and decreased the mitochondrial permeability transition pore opening, thereby improving mitochondrial function.

**Conclusion:** Pretreatment with Cap can regulate autophagy by upregulating 14-3-3γ expression, inhibiting oxidative stress and inflammation, maintaining mitochondrial function, and protecting cardiomyocytes against LPS-induced injury.

## Introduction

Sepsis is characterized by high mortality in intensive care units (Angus et al., 2006; Schorr et al., 2014)**.** Lipopolysaccharide (LPS), an essential component of the gram-negative bacterial outer membrane, is recognized as the leading cause of multi-organ failure in sepsis (Tsiotou et al., 2005; Zanotti-Cavazzoni Hollenberg, 2009). LPS-induced sepsis occurs due to excessive release of inflammatory cytokines, overproduction of oxygen radicals, and mitochondrial dysfunction, and it is a major contributor to cardiac dysfunction (Li et al., 2016; Okuhara et al., 2017; Liu et al., 2019). Mitochondria in cardiomyocytes not only generate ATP but also modulate oxidative stress, signaling, and cell fate (Kolwicz et al., 2013). Mitochondrial impairment is correlated with overproducing intracellular/mitochondrial reactive oxygen species (ROS) and inflammatory responses during LPS-induced sepsis in cardiomyocytes (Zang et al., 2007; Zang et al., 2010; Sun et al., 2018).

Accumulating studies indicated that organelles damaged by LPS-induced sepsis (such as mitochondria) were cleared by the activation of autophagy (Hsiao et al., 2012; Lin et al., 2014). Autophagy protects cardiomyocytes during LPS-induced sepsis by activating AMP-activated protein kinase (AMPK) pathways (Nishida et al., 2009). AMPK is a central energy sensor in eukaryotes that responds to disequilibrium of AMP/ADP (Lee et al., 2010; Roach, 2011). Besides, 14-3-3 proteins are evidence that it interacts directly or indirectly with multiple molecules, including AMPK, mammalian targets of rapamycin (mTOR), and Unc-51-like autophagy activating kinase 1 (ULK1), to regulate the autophagy process (Ma et al., 2012). 14-3-3γ, an isoform of the 14-3-3 protein family, was demonstrated in our previous studies to offer a cardioprotective role in response to LPS-induced cardiotoxicity (He et al., 2006; Liu et al., 2014; Huang et al., 2018a). However, the specific mechanism of 14-3-3γ-dependent autophagy remained unclear and further exploration in in cardiomyocyte LPS-induced sepsis was needed.

Several studies have confirmed many pharmacological agents as potential preconditioning strategies to prevent myocardial injury (Hausenloy and Yellon, 2011). Our previous studies suggested that pretreatment with astragaloside Ⅳ or tanshinone ⅡA could elicit similar protective effects as ischemic preconditioning against anoxia/reoxygenation (A/R)-induced cardiomyocyte injury (Zhang et al., 2018; Luo et al., 2019). Capsaicin (Cap) targets multiple pathways and possesses many pharmacological properties, including antimicrobial, analgesic, antiinflammation and antioxidant (Sun et al., 2015; Fernandes et al., 2016; Gerber et al., 2019). In our previous study, pretreatment with Cap improved cardiac function via 14-3-3η or SIRT1/Bcl2 following A/R injury (He et al., 2017; Huang et al., 2018a; Qiao et al., 2020). However, the effect of Cap pretreatment on LPS-induced cardiotoxicity remains unclear. Here, we used the LPS-induced sepsis model in neonatal rat cardiomyocytes (NRCMs), to explore (1) the role of Cap during 14-3-3γ-related autophagy process via the AMPK-mTOR/ULK1 pathway in LPS-challenged cardiomyocytes; (2) the effects of Cap in LPS-induced cardiotoxicity via regulating inflammatory cytokine release, oxidative stress, and mitochondrial dysfunction.

## Materials and Methods

### Materials

The following reagents were purchased: LPS, from Sigma-Aldrich (St. Louis, MO, USA); Cap (purity ≥ 98%), from the National Institutes for Food and Drug Control (Beijing, China); adenovirus pAD/14-3-3γ-shRNA, from Gene Chem Co., Ltd (Shanghai, China); bafilomycin A1 (BafA1) and compound C, from Sigma-Aldrich (St. Louis, MO, USA); antibodies against 14-3-3γ, P62, NADH dehydrogenase [ubiquinone] 1 beta subcomplex subunit 8 (NDUFB8) and cytochrome b-c1 complex subunit 2 (UQCRC2), from Abcam (Cambridge, UK). Anti-LC3, -AMPKα, -phospho-AMPK (phosphorylation at Ser172), -mTOR, -phospho-mTOR (phosphorylation at Ser2448), -ULK1, -phospho-ULK1 (phosphorylation at Ser757) antibodies, from Cell Signaling Technology (Beverly, MA, USA), and horseradish peroxidase-conjugated IgG secondary antibody, from Zsbio (Beijing, China).

### Primary culture of neonatal rat cardiomyocytes (NRCMs) and adenoviral infection equations

All experimental procedures were performed according to the Guide for the Care and Use of Laboratory Animals published by the US National Institutes of Health (NIH Publication no. 85–23, revised 1996) and approved by the Ethics Committee of Nanchang University (No. 2019–0036). The NRCMs from 0–3 days-old Sprague-Dawley rats (the Animal Center of Nanchang University, Nanchang, China) were prepared as previously published (He et al., 2017). Cardiomyocytes were cultured in high-glucose Dulbecco’s modified Eagle medium (DMEM, Gibco-BRL, Grand Island, NY, USA) supplemented with 20% fetal bovine serum (FBS, Gibco-BRL), 100 U/ml of penicillin and streptomycin, and 1% bromodeoxyuridine (Brdu, Solarbio Science & Technology, Beijing, China), and incubated in a 95% air and 5% CO_2_ humidified atmosphere incubator at 37 °C.

Cardiomyocytes were infected by adenovirus pAD/14-3-3γ-shRNA and infection efficiency was approximately 85% after 48 h (Qiao et al., 2020). Before conducting subsequent experiments, the transfected cardiomyocytes were cultured in 95% O_2_ and 5% CO_2_ for 12 h at 37 °C.

### Experimental grouping and reagent treatment

#### Phase A

First, we investigated whether Cap could protect cardiomyocytes against LPS-induced injury. Cardiomyocytes were randomly distributed into four groups: LPS, Cap, pAD/14-3-3γ-shRNA, and control. Cells in the control group were cultured in a complete medium throughout the experiments. LPS grouped-cardiomyocytes were treated with 1 mg/L LPS for 24 h (Liu et al., 2014; Huang et al., 2018a). Then, the cells in the Cap group were pretreated with 5, 10, 20, 40, 80 μM Cap for 12 h, placed in the fresh culture medium, and then exposed to 1 mg/L LPS for 24 h. Cardiomyocytes in the pAD/14-3-3γ-shRNA group were treated with pAD/14-3-3γ-shRNA for 12 h before Cap pretreatment. Cell viability, LDH and CK activities, and 14-3-3γ expression were determined after processing.

#### Phase B

Next, we examined the changes in autophagy in LPS-induced cardiomyocyte injury and the effects of Cap on these changes. Cardiomyocytes were cotreated with or without 100 nM BafA1, an autophagy inhibitor (Mauthe et al., 2018), and LPS for 24 h. The expression of LC3 and P62 were determined after processing. After, cardiomyocytes were pretreated with 10 μM Cap for 12 h according to the method of phase A before LPS treatment. Cardiomyocytes in the control, LPS, and pAD/14-3-3γ-shRNA group were treated according to phase A. The expressions of 14-3-3γ, LC3, and P62, along with autolysosome contents were determined again after processing.

#### Phase C

Furthermore, we explored the role of the AMPKα/mTOR signaling pathway in LPS-induced cardiomyocyte injury. Cardiomyocytes were randomly distributed into four groups: control, LPS, compound C, and Cap. Cardiomyocytes in the control, LPS, and Cap groups were treated similarly as mentioned above. Cardiomyocytes in the compound C group were coincubated with 5 μM compound C (AMPK inhibitor, Dasgupta and Seibel, 2018) and 10 μM Cap for 12 h according to phase A before LPS treatment. The expressions of 14-3-3γ, LC3, P62, AMPKα, AMPK phospho-Ser172, mTOR, mTOR phospho-Ser2448, ULK1, and ULK1 phospho-Ser757 were then determined .

#### Phase D

Finally, we studied how LPS disrupts intracellular redox equilibrium and cytokines and impairs mitochondrial function. In brief, the control, LPS, Cap, and pAD/14-3-3γ-shRNA group were treated as in phase A. Intracellular/mitochondrial ROS, the activities of GSH-Px, SOD, catalase, MDA, and ATP levels, GSH/GSSG ratio, cytokine (IL-1β, TNF-α, IL-6, IL-10) contents, mitochondrial membrane potential (MMP), and mitochondrial permeability transition pore (mPTP) opening were determined at the end of experiments.

### Measurement of cell viability and biochemical parameters

Cell viability was measured using a commercially available kit (CCK-8, TransGen Biotech, Beijing, China). In brief, cells were cultured in 96-well plates at a density of 3× 10^3^ cells/well and treated as in phase A. Then, we added the tetrazolium salt WST-8 to the medium at a certain ratio incubated for 1–2 h at 37 °C, and measured the number of viable cells using a microplate reader (Bio-Rad 680, Hercules, CA, USA).

The culture medium was collected after LPS challenge to examine lactate dehydrogenase (LDH) and creatine phosphate kinase (CK) activities using commercially available kits (Jiancheng, Nanjing, China, Qiao et al., 2020).

### Measurements of inflammatory cytokines

The culture medium was collected after LPS challenge and centrifuged for 10 min at 3000 rpm, and the inflammatory cytokine (IL-1β, TNF-α, IL-6, and IL-10) levels were measured by enzyme-linked immunosorbent assay (ELISA) (BestBio, Shanghai, China).

### Measurements of intracellular/mitochondrial ROS

Intracellular/mitochondrial ROS levels were detected using the oxidation-sensitive probe (DCFH-DA or Mito-SOX) as previously described (Zuo et al., 2018). After treatment as in phase A, cardiomyocytes were harvested and incubated with DCFH-DA (Beyotime, Shanghai, China) or Mito-SOX (Invitrogen™ Oregon, USA) in the dark at 37 °C. Then, fluorescence was detected by a flow cytometer (Beckman Coulter, Brea, CA, USA).

Moreover, intracellular/mitochondrial ROS intensity was observed under a fluorescence microscope (×100 magnification, Olympus, Japan). In brief, cells were seeded in 24-well plates at a density of 1×10^4^ cells/coverslip, then washed with prewarmed PBS and incubated with the fluorescence dye dihydroethidium (DHE, BestBio, Shanghai, China) or Mito-SOX in the dark at 37 °C.

### Measurement of endogenous antioxidant enzyme activities and glutathione (GSH) and glutathione disulfide (GSSG)

The endogenous antioxidant enzyme activities, including glutathione peroxidase (GSH-Px), superoxide dismutase (SOD), and catalase; levels of malondialdehyde (MDA) (Jiancheng, Nanjing, China); and contents of the nonenzymatic antioxidant system (GSH, GSSG, and GSH/GSSG ratio; Beyotime, Shanghai, China) were detected by spectrophotometry, respectively (Chen et al., 2020; Qiao et al., 2020). Cells were lysed after treatment as in phase A, and the supernatant was collected and examined based on manufacturer instructions of commercial kits.

### Dansylcadaverine (MDC) and LysoTracker Red Staining

Cells were seeded in 24-well plates at a density of 1×10^4^ cells/coverslip and treated as described in phase A. Then, cardiomyocytes were washed with prewarmed PBS and incubated with fluorescence dye MDC (Beyotime, Shanghai, China) or LysoTracker Red DND-99 (Invitrogen™, Oregon, USA) in darkness for 30 min at 37 °C. After that, images were captured by a fluorescence microscope (Olympus, Japan).

### Western blotting

Proteins were extracted by a protein extraction kit (Applygen Technologies Inc, Beijing, China), and quantified by a bicinchoninic acid (BCA) protein assay kit (Thermo Fisher, Massachusetts, USA). Equal amounts of protein (30 μg) were separated by SDS-PAGE and later transferred to a polyvinylidene fluoride (PVDF) membrane. The PVDF membrane was then blocked with 5% bull serum albumin, and subsequently incubated with primary antibodies (14-3-3γ, LC3, P62, AMPKα, p-AMPKα, mTOR, p-mTOR, ULK1, p-ULK1, NDUFB8, UQCRC2, and β-actin) overnight at 4 °C. The membranes were blotted with horseradish peroxidase conjugated secondary antibody and immersed with an enhanced-chemiluminescence substrate. Finally, protein bands were imaged and analyzed with the Quantity One software (Bio-Rad, USA, Qiao et al., 2020).

### ATP production

Intercellular ATP levels were measured using an Enhanced ATP Assay Kit (Beyotime, Shanghai, China). Cells were lysed and the supernatant was collected. It was then added to a detecting solution in a lightproof 96-well plate and incubated for 5 min at 25 °C. Total cellular ATP levels were determined from real-time luminescence signals and were normalized to the protein concentrations (Li et al., 2017).

### Measurement of MMP and mPTPs openness

MMP was detected by Fluorescent probe JC-1 (BestBio, Shanghai, China). Briefly, cells were harvested by trypsin without EDTA and incubated with JC-1 in the dark for 30 min at 37 °C. Then, cells were suspended in an incubation buffer and MMP measured by flow cytometer (Beckman Coulter, Brea, CA, USA) at 530/580 nm (red) and 485/530 nm (green). The ratio of Red:green fluorescence intensity represents the MMP level (Qiao et al., 2020).

The mPTP opening was examined as described previously (He et al., 2020). The carmidiocyte mitochondria were isolated using a mitochondrial isolation kit (Thermo Fisher, Massachusetts, USA). Afterward, fractions were resuspended in 160 μl swelling buffer (KCl 120 mM, Tris-HCl 10 mM, MOPS 20 mM, KH_2_PO_4_ 5 mM), the suspensions plated onto a 96-well microtiter plate and florescence measured at 520 nm. Then, we added a 40-μl CaCl_2_ solution (200 nM) to stimulate the mPTP openings. The absorbance values were calculated per minute at 520 nm until the trends stabilized. The degree of mPTP openness were determined by the extent of changes at 520 nm.

### Statistical Analysis

All experiments values were represented as Mean ± S.E.M., and tested by one-way ANOVA, the differences of biochemical data between each group were further tested by Tukey's honestly significant difference test. *P* < 0.05 was considered to be statistically significant.

## Results

### Cap protects cardiomyocytes by upregulating 14-3-3γ expression against LPS challenge

**FIGURE 1:**
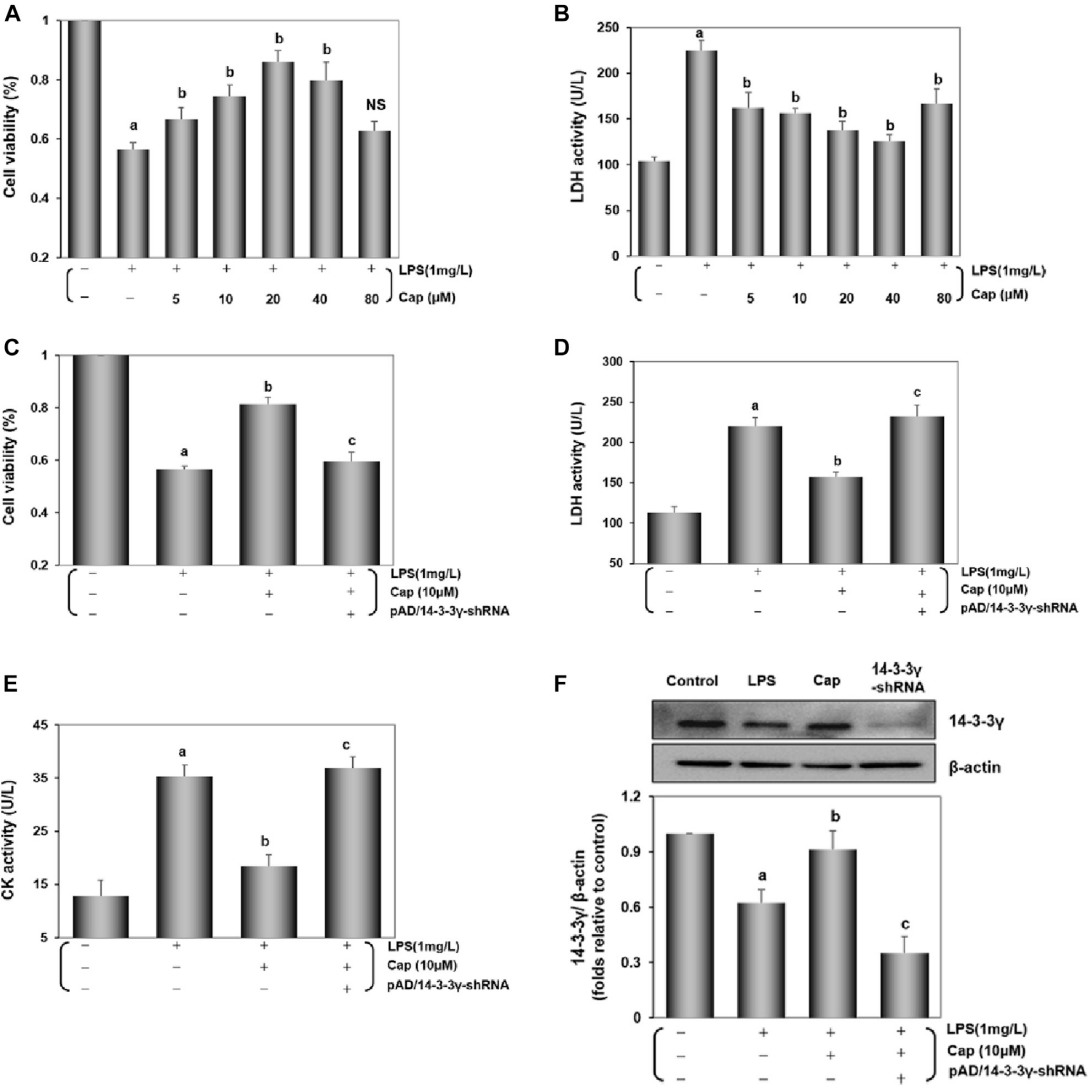
Cap protects cardiomyocytes by upregulating 14-3-3γ levels after LPS challenge. **(A)** and **(B)** Cap presented a concentration-dependent effect on cell viability and LDH activity against LPS-induced cardiomyocyte injury. **(C)** Cap increased cell viability, and cotreatment with pAD/14-3-3γ-shRNA nullified Cap effects after LPS challenge. **(D)** and **(E)** LDH and CK activity in culture medium. **(F)** The representative western blot bands and the relative band intensity of 14-3-3γ expression in cardiomyocytes. Values are presented as mean ± SEM. For five individual experiments, a: *P*<0.01 vs. the control group; b: *P*<0.01 vs. the LPS group; c: *P*<0.01 vs. the Cap+LPS group.

Cardiomyocytes were pretreated with Cap (0 μM-80 μM) for 12 h and again coincubated with 1 mg/L LPS for 24 h. Cap caused a concentration-dependent increase in cell viability and decrease in LDH activity; the optimal concentration of Cap was 10 μM (Figures 1A,B). Cap increased cell viability and inhibited the leakage of LDH and CK in response to LPS-induced injury significantly (*P* < 0.01); however, these positive effects were nullified by pAD/14-3-3γ-shRNA (*P* < 0.01, Figures 1C–E). Besides, 14-3-3γ expression was significantly decreased in the LPS group compared to that in the control group (*P* < 0.01), whereas Cap upregulated 14-3-3γ expression (*P* < 0.01, Figure 1F).

Together, these data indicated that LPS toxicity could downregulate 14-3-3γ expression and trigger cardiomyocyte injury, but pretreatment with Cap could upregulate the 14-3-3γ protein and alter the cell viability plus LDH and CK activities to protect cardiomyocytes.

### Cap activates cardiomyocyte autophagy against LPS-induced injury

**FIGURE 2:**
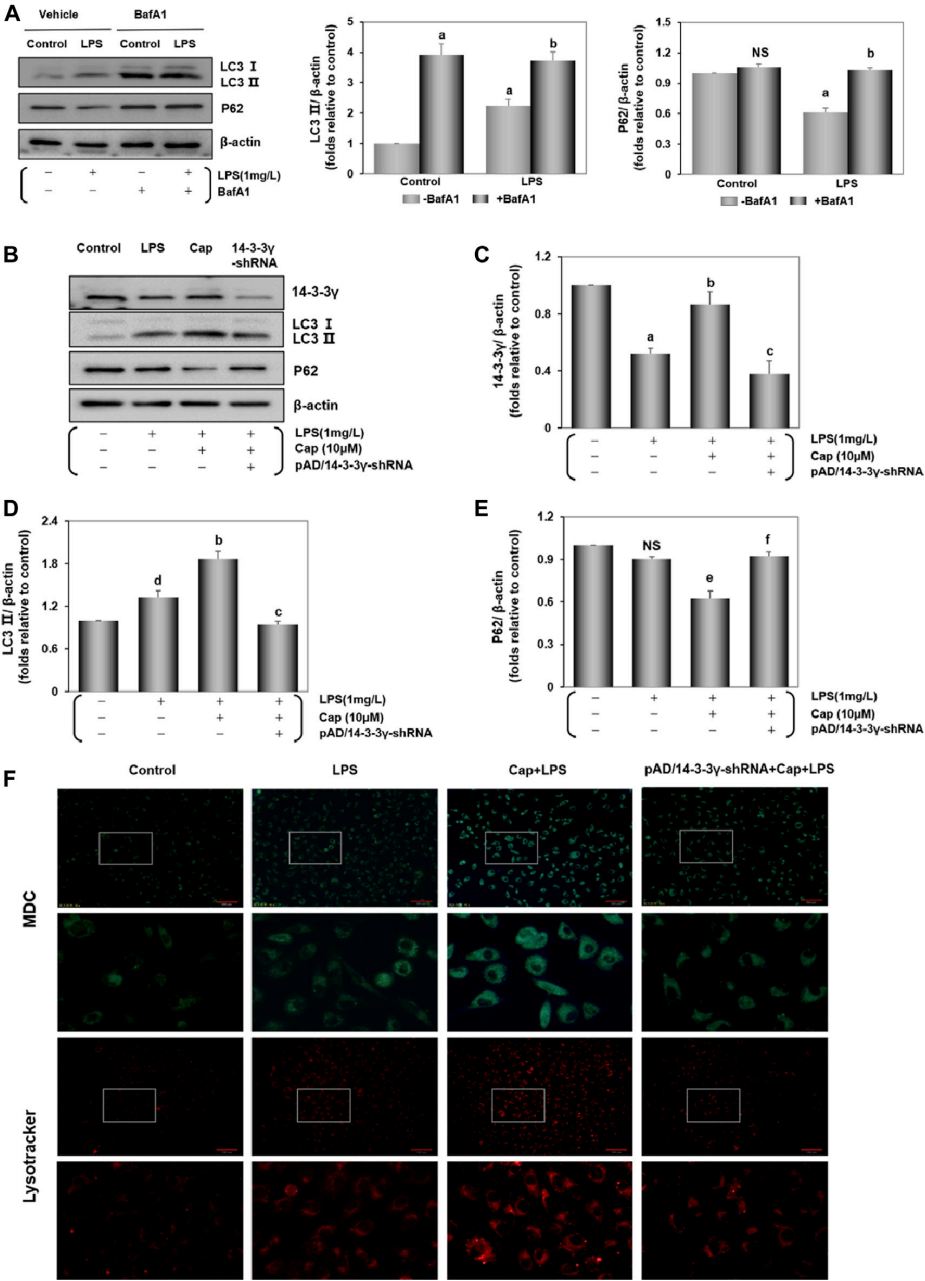
Cap activates cardiomyocyte autophagy against LPS-induced injury. **(A)** LPS-induced autophagy flux confirmed by the contrast of P62 and LC3 Ⅱ between cells with and without BafA1 addition. **(B)** The representative western blot bands of 14-3-3γ, LC3 Ⅱ, and P62 expression in the cardiomyocytes. **(C-E)** The relative band intensity of 14-3-3γ, LC3 Ⅱ, and P62 expression in the cardiomyocytes. **(F)** Cardiomyocytes incubated with dansylcadaverine (MDC) or LysoTracker Red DND-99. Green or red intensity showed the autophagosome and autolysosomes. Values were presented as mean ± SEM. For five individual experiments, a: *P*<0.01 vs. the control group; b: *P*<0.01 vs. the LPS group; c: *P*<0.01 vs. the Cap+LPS group. d: *P*<0.05 vs. the control group; e: *P*<0.05 vs. the LPS group; f: *P*<0.05 vs. the Cap+LPS group; NS, nonsignificant.

Previous studies documented the role of autophagy in LPS-induced cardiac injury (Sun et al., 2018; Huang et al., 2020). The levels of LC3Ⅱ, an indicator of autophagy levels, and the expression of P62, an autophagic substrate protein (Ma et al., 2012), were increased (*P* < 0.01) and decreased (*P* < 0.01), respectively, after LPS challenge (Figure 2A). To identify LC3Ⅱ changes, which reflected as the increase in autophagy levels rather than the impairment of autophagic flux, cardiomyocytes were treated with BafA1-an inhibitor that blocks the alternation of autophagosomes to autolysosomes (Yamamoto et al., 1998). Figure 2A showed that BafA1 induced an increase in LC3Ⅱ and P62 levels in the control and LPS group.

Unlike in the LPS group, pretreatment with Cap led to significant LC3Ⅱ accumulation (*P* < 0.01) and P62 (*P* < 0.05) led to the reverse; however, these responses were nullified by pAD/14-3-3γ-shRNA (Figures–E).

Furthermore, the acidotropic dyes can stain intracellular acid compartments. MDC is used in labeling early autophagosomes (Bampton et al., 2005; Wang et al., 2020) and Lyso Tracker Red is a marker for the later stages of autophagy (Scott et al., 2004). As illustrated in Figure 2F, MDC-specific dots (green) and autolysosome signals (red) were detected after LPS challenge, and both fluorescence intensities were significantly enhanced with Cap pretreatment. Moreover, coincubation with pAD/14-3-3γ-shRNA reduced the green and red dots. These results corroborate the above the data on LC3Ⅱ and P62 expression.

Taken together, these results indicated that LPS induced an accumulation of LC3Ⅱ to active autophagy; pretreatment with Cap could further enhance the autophagic flux to scavenge the misfolded proteins and dysfunctional cellular components. Conversely, these protective effects were nullified by coincubation with pAD/14-3-3γ-shRNA.

### Cap upregulates 14-3-3γ and integrates AMPKα/mTOR pathway against LPS-induced injury

**FIGURE 3:**
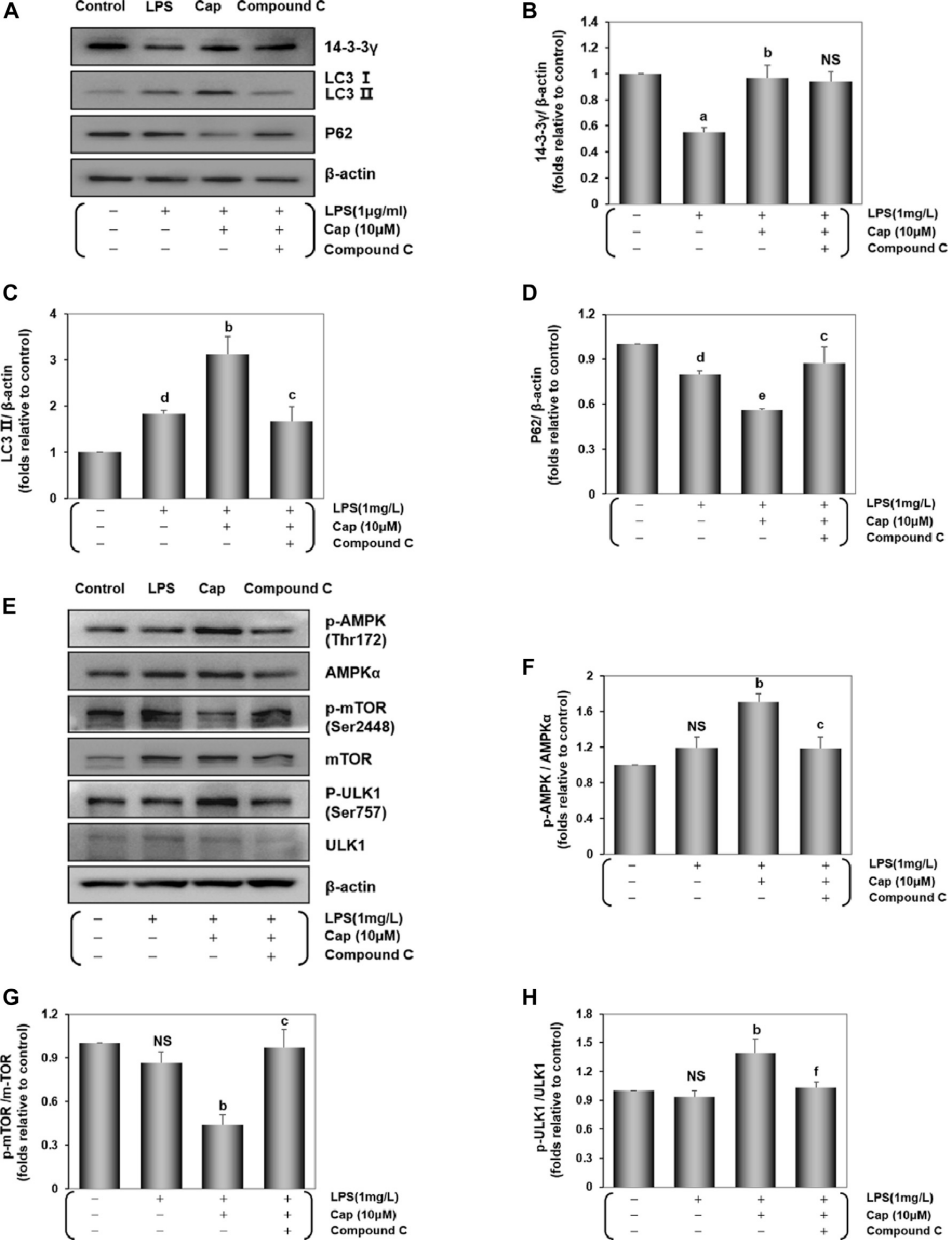
Cap upregulates 14-3-3γ and integrates AMPKα/mTOR pathway against LPS-induced injury. **(A)** The representative western blot bands of 14-3-3γ, LC3 Ⅱ, and P62 expression in the cardiomyocytes. **(B-D)** The relative band intensity of 14-3-3γ, LC3 Ⅱ, and P62 expression in the cardiomyocytes. **(E)** The representative western blot bands of total and phosphorylated mTOR, AMPK and ULK1 expression in the cardiomyocytes. **(F-H)** The relative band intensity of p-mTOR/mTOR, p-AMPK/AMPK, and p-ULK1/ULK1 expression in the cardiomyocytes. Values are presented as mean ± SEM. For five individual experiments, a: *P*<0.01 vs. the control group; b: *P*<0.01 vs. the LPS group; c: *P*<0.01 vs. the Cap+LPS group. d: *P*<0.05 vs. the control group; e: *P*<0.05 vs. the LPS group; f: *P*<0.05 vs. the Cap+LPS group; NS, nonsignificant.

The 14-3-3 protein influences the autophagy process (Lee et al., 2020). We investigated the underlying molecular pathway involved in 14-3-3γ regulation of autophagic levels. As shown in Figures 3A–D **,** compared with the LPS group, pretreatment with Cap significantly increased 14-3-3γ (*P* < 0.01) and LC3Ⅱ (*P* < 0.01) expression, but decreased P62 (*P* < 0.05) expression. Interestingly, cotreatment with Cap and Compound C, an inhibitor of AMPK, upregulated 14-3-3γ (NS, nonsignificant vs. Cap+LPS), increased P62 expression (*P* < 0.01 vs. Cap+LPS), but reduced LC3Ⅱ (*P* < 0.01 vs. Cap+LPS) after LPS challenge, indicating that the effects of 14-3-3γ upregulation by Cap during LPS-mediated autophagy might be related to AMPK.

Then, we examined changes in the expressions of AMPKα, mTOR, a common negative factor of autophagy (Kim and Guan, 2015), and ULK1. The p-AMPK/AMPKα and p-ULK1/ULK1 ratio in the Cap-pretreated cardiomyocytes were higher than in the LPS group (*P* < 0.01, Figures 3E,F,H), while p-mTOR expression was lower than that in the LPS group (*P* < 0.01, Figures 3E,G). Conversely, active phosphorylation sites of AMPK and ULK1, plus mTOR levels were reversed by Compound C addition.

These results demonstrated that Cap pretreatment involved a positive adjustment in autophagy level by upregulating 14-3-3γ, AMPK, and ULK1 levels, and suppressing mTOR expression. The results also demonstrated that the underlying mechanism of 14-3-3γ-dependent autophagy might be related with AMPK activity after LPS-induced cardiomyocyte injury.

### Cap inhibits the activation of inflammatory cytokines in LPS-challenged cardiomyocytes

**FIGURE 4:**
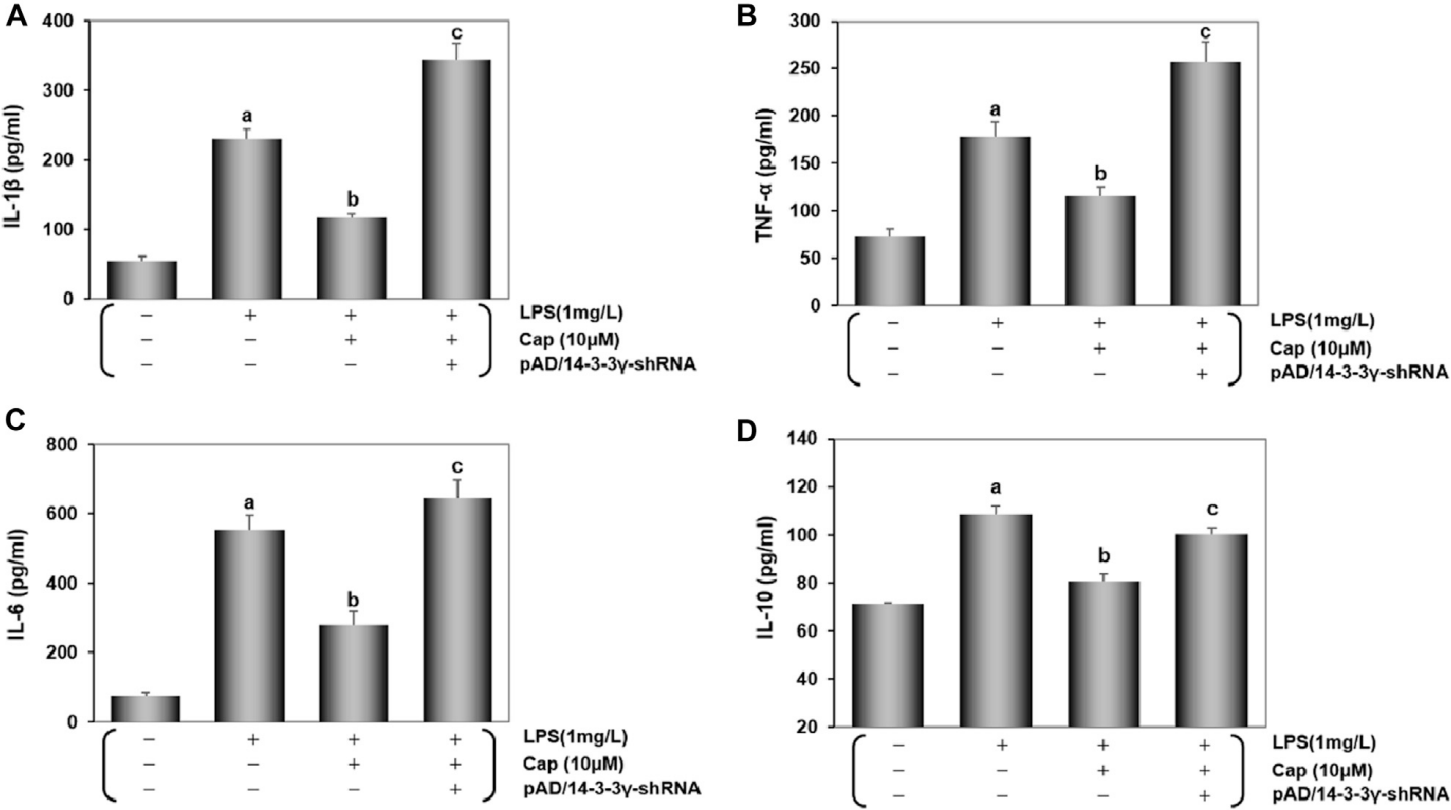
Cap inhibits the activation of inflammatory cytokines in LPS- challenged cardiomyocytes. **(A-D)** IL-1β, TNF-α, IL-6, and IL-10 were measured in the culture supernatant of cardiomyocytes by diversiform ELISA assays. Values are presented as mean ± SEM. For five individual experiments, a: P<0.01 vs. the control group; b: P<0.01 vs. the LPS group; c: P<0.01 vs. the Cap+LPS group.

As an important index of LPS-induced damage (Schwerd et al., 2017), cytokine activities in cardiomyocytes were measured by ELISA (Figure 4). Contrasted with normal cardiomyocytes, IL-1β, TNF-α, IL-6, and IL-10 levels were significantly increased after LPS-induced injury (*P* < 0.01), but this index levels declined significantly with Cap pretreatment (*P* < 0.01). In contrast, the protective effects of Cap were weakened by adding pAD/14-3-3γ- shRNA (*P* < 0.01).

These results were consistent with Cap protecting cardiomyocytes from LPS-induced sepsis by upregulating 14-3-3γ expression.

### Cap inhibits oxidative stress in injured cardiomyocytes after LPS challenge

**FIGURE 5:**
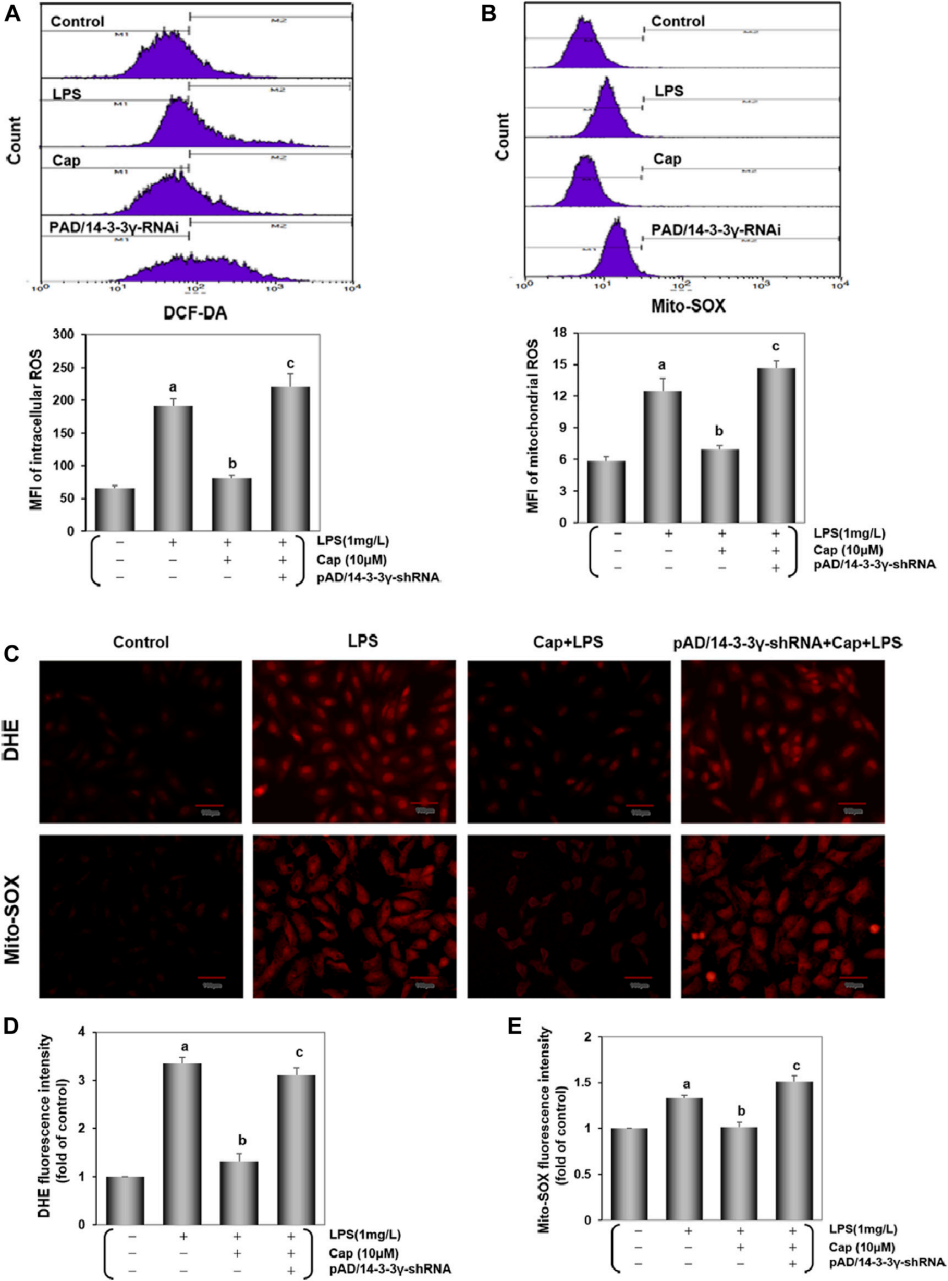
Cap decreases Intracellular/mitochondrial ROS generation in cardiomyocytes injured after LPS challenge. **(A)** DCF-DA indicated intracellular ROS generation. **(B)** Mito-SOX measured mitochondrial ROS level. **(C)** Intracellular/ mitochondrial ROS were stained by DHE/Mito-SOX probe. **(D)** and **(E)** Fluorescence intensity of DHE and Mito-SOX were analyzed by ImageJ software. Values were presented as mean ± SEM. For five individual experiments, a: *P*<0.01 vs. the control group; b: *P*<0.01 vs. the LPS group; c: *P*<0.01 vs. the Cap+LPS group.

Accumulating studies demonstrated that excessive ROS generation could induce cardiac inflammation following LPS challenge (Suzuki et al., 2012; Zhao et al., 2016). Intracellular/mitochondrial ROS were detected by flow cytometry and fluorescence microscopy. As shown in Figures 5A,B, after adding LPS 24 h, the intracellular/ mitochondrial ROS curve moved significantly toward the right and the peak value was progressively enhanced (*P* < 0.01), while the change was inverted with Cap pretreatment (*P* < 0.01). The addition of pAD/14-3-3γ-shRNA nullified the protective effect of Cap (*P* < 0.01). Consistent with the results above, in LPS-challenged cardiomycetes, intense fluorescent dots were observed under fluorescence microscopy using DHE or Mito-SOX, but pretreatment with Cap reduced the fluorescence intensity (Figures 5C–E).

**FIGURE 6:**
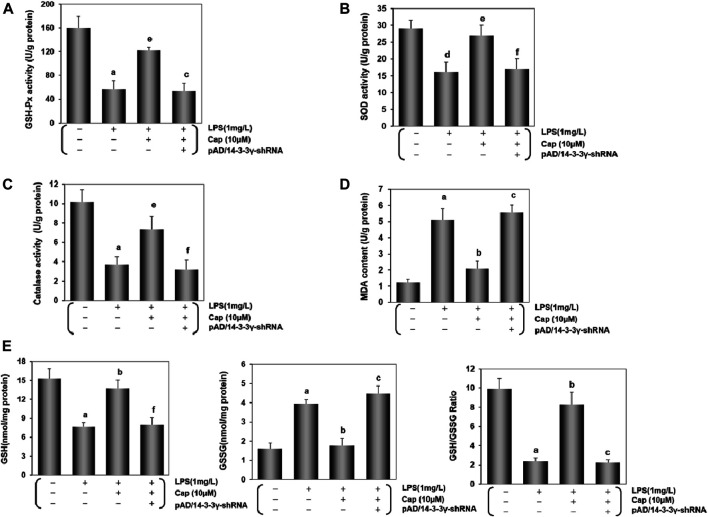
Cap inhibits oxidative stress in injured cardiomyocytes after LPS challenge. **(A-D)** Activities of GSH-Px, SOD, and catalase, and MDA content in cardiomyocytes after different treatments. **(E)** Levels of intracellular glutathione in the cardiomyocyte. Left: intracellular GSH levels; middle: intracellular GSSG levels; right: intracellular GSH/GSSG ratio. Values are presented as mean ± SEM. For five individual experiments, a: *P*<0.01 vs. the control group; b: *P*<0.01 vs. the LPS group; c: *P*<0.01 vs. the Cap+LPS group. d: *P*<0.05 vs. the control group; e: *P*<0.05 vs. the LPS group; f: *P*<0.05 vs. the Cap+LPS group.

To confirm the change of oxidative status in cardiomyocytes, we examined the activities of endogenous antioxidant enzymes (GSH-Px, SOD, and catalase), lipid metabolite content (MDA), and non-enzymatic antioxidant system levels (GSH, GSSG and GSH/GSSG). Contrasted with the control group, the activities of GSH-Px, SOD and catalase exhibited a sharp decline while MDA contentshowed a progressive increase in the LPS-treated group **(** *P* < 0.01, FiguresA–D). Pretreatment with Cap reversed these indices to scavenge oxygen radicals in the cardiomyocytes, but the mentioned beneficial effects on the cardiomyocytes were offset after deregulating 14-3-3γ expression. Moreover, GSH and GSH/GSSG decreased, while GSSG exhibited the reverse trend after LPS-induced toxic effects (*P* < 0.01, Figure 6E). Pretreatment with Cap could protect the cardiomyocytes from LPS-induced damage by excessive oxygen species, but pAD/14-3-3γ-shRNA addition could aggravate the damage induced by LPS.

These data demonstrated that LPS generated excessive ROS and decreased the scavenging of oxygen radicals. However, pretreatment with Cap could protect the cardiomyocytes from reducing oxidative stress by upregulating 14-3-3γ expression.

### Cap maintains mitochondrial function by regulating mitochondrial bioenergetics in cardiomyocytes after LPS challenge

**FIGURE 7:**
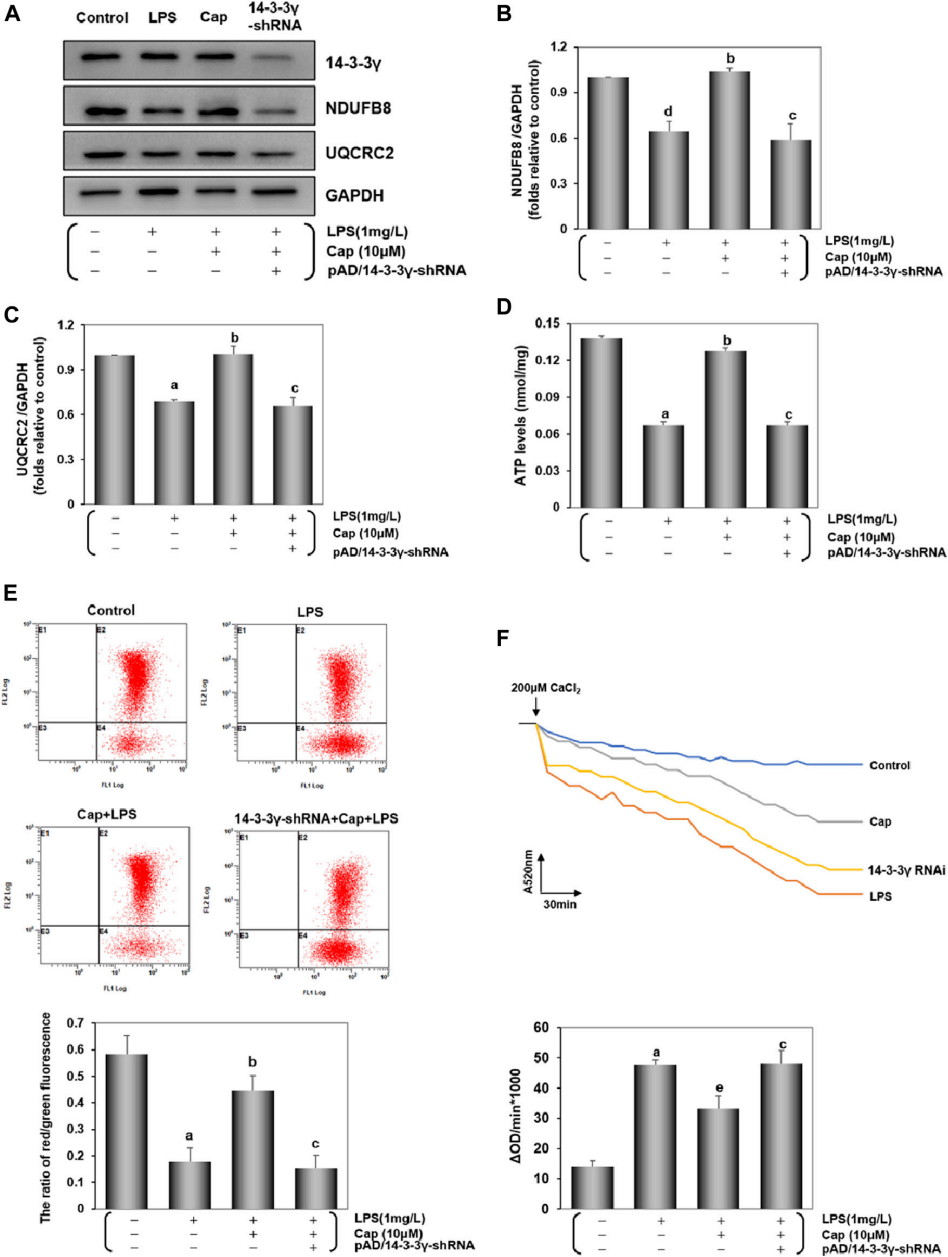
Cap maintains mitochondrial function by regulating mitochondrial bioenergetics in cardiomyocytes after LPS challenge. **(A)** The representative western blot bands of NDUFB8 and UQCRC2 expression in the cardiomyocytes. **(B)** and **(C)** The relative band intensity of NDUFB8 and UQCRC2 expression in the cardiomyocytes. **(D)** Cardiomyocyte ATP levels were measured by firefly luciferase assay. **(E)** Levels of MMP were detected by JC-1, and red/green fluorescence ratio indicated MMP levels. **(F)** Mitochondrial swelling by Ca^2+^-induced cells was used for measuring mPTP opening. The changes in absorbance values at 520 nm per minute were recorded and the mPTP opening levels are shown by fluctuations in absorbance (ΔOD = A520_0min_-A520_30min_). Values are presented as mean ± SEM. For five individual experiments, a: *P*<0.01 vs. the control group; b: *P*<0.01 vs. the LPS group; c: *P*<0.01 vs. the Cap+LPS group. d: *P*<0.05 vs. the control group; e: *P*<0.05 vs. the LPS group.

Studies have suggested that mitochondrial dysfunction via LPS toxicity triggers cardiomyocytes damage (Zhou et al., 2011; Liu et al., 2018). The expression of NDUFB8 and UQCRC2 can reflect mt complex Ⅰ/Ⅲ activities. Comparison of NDUFB8 or UQCRC2 expression between the control and LPS groups (Figures 7A–C) revealed that NDUFB8 (*P* < 0.05) and UQCRC2 (*P* < 0.01) expressions were decreased after LPS challenge, indicating an impairment of the mitochondrial electron transport chain (ETC) following LPS-induced injury. Meanwhile, LPS induced a significant decrease in ATP levels (*P* < 0.01, Figure 7D). Pretreatment with Cap upregulated NDUFB8 and UQCRC2 (*P* < 0.01, Figures 7B,C), and restored ATP levels (*P* < 0.01), but these positive effects were nullified by adding pAD/14-3-3γ- shRNA.

The alternation of MMP and mPTP opening served as biological markers for the mitochondrial function (Dai et al., 2020; Kinnally et al., 2011). The ratio of red/green fluorescence represents the degree of MMP oscillation in cells. Compared with the control group, the decline in the red/green ratio reflected a loss of MMP in the LPS group, whereas Cap-pretreatment restored the cardiomyocytes from MMP loss (*P* < 0.01, Figure 7E). Additionally, Figure 7F shows that the mPTP opening was activated following LPS challenge unlike in the control group (*P* < 0.01); however, pretreatment with Cap presented a steady downward trend (*P* < 0.05). The above protective effects could be blunted when 14-3-3γ expression was significantly reduced using pAD/14-3-3γ-shRNA.

From these results, we discovered that LPS-induced toxicity could trigger mitochondrial damage in the cardiomyocytes. Nevertheless, pretreatment with Cap effectively improved the mitochondrial function by upregulating the 14-3-3γ protein.

## Discussion

The study explored whether (i) upregulating 14-3-3γ via Cap pretreatment contributed to autophagy via the AMPK-mTOR/ULK1 pathway against LPS-induced sepsis to myocardial injury (ii) Cap protected cardiomyocytes from LPS-induced inflammation, excessive oxidative stress, and mitochondrial dysfunction. Accumulating studies indicated that LPS triggered cellular damage, especially in cardiomyocytes (Drosatos et al., 2013; Beesley et al., 2018). Here, we found that cell viability was significantly decreased and the LDH and CK activities were increased following LPS stimulation (Figures 1C–E). Interestingly, Cap presented a concentration-dependent increase to protect cardiomyocytes in response to LPS-induced cardiotoxicity (Figures 1A,B), indicating a beneficial role in cardiomyocytes following damage.

Our previous studies have demonstrated that Cap has multiple targets, including 14-3-3η and SIRT1, by which it protects cardiomyocytes against anoxia/reoxygenation-induced damage (Qiao et al., 2020; Huang et al., 2018a; He et al., 2017). In the study, Cap-mediated positive effects on cardiomyocytes were related to 14-3-3γ expression after LPS-induced injury (Figures 1–7). 14-3-3γ (an isoform of the 14-3-3 family proteins in mammals) was evidenced by its involvement in variable cellular processes, for example cell proliferation (Kumar et al., 2003), survival (Masters et al., 2002) and apoptosis (Zha et al., 1996). In the previously published work, we focused on 14-3-3γ/Bcl-2-mediated apoptotic pathway regulation in cardiomyocytes or human umbilical vein endothelial cells (HUVECs) against LPS or doxorubicin toxicity (He et al., 2006; Liu et al., 2014; Huang et al., 2018b; Yang et al., 2019). The specific mechanism of 14-3-3γ upregulation via Cap pretreatment remained unclear, as well as the effects by other programmed cell death pathways in cardiomyocytes after LPS-induced injury were also worth studying.

Autophagy-dependent cell death, which is driven by autophagy-related genes, significantly affects LPS-induced cardiotoxicity and ischemia/reperfusion-induced injury in cardiomyocytes (Ma et al., 2012; Tang et al., 2019; Di et al., 2020). In this study, the level of LC3Ⅱ increased and P62 presented reversed trend in cardiomyocytes following the LPS challenge. Besides, after coincubation with BafA1, LC3Ⅱ and P62 levels were significantly improved, indicating that LPS treatment could trigger a low level of autophagy instead of impairment in autophagic flux (Figure 2A). Previous studies have suggested that activation of autophagy could contribute to suppressing LPS-induced cell toxicity (Sun et al., 2018; Quach et al., 2019; Wu et al., 2020), autophagy influences the clearance of damaged proteins and organelles (Yang and Klionsky, 2010). After pretreatment with Cap, LC3Ⅱ was significantly increased and P62 degradation was declined (Figures 2B–E). Furthermore, autophagosomes, autolysosomes, and lysosomes were increased in Cap-pretreated cardiomyocytes (Figure 2F). These results correlated with the above data and suggested that Cap-mediated protection effects on cardiomyocytes might be related to activating autophagy.

AMPK is a well-known energy sensor in eukaryotes (de Pablos et al., 2019; Mihaylova and Shaw, 2011), and is also a key regulator in multiple cellular processes, such as ferroptosis (Lee et al., 2020), pyroptosis (He et al., 2016), and autophagy (Wang et al., 2018). Interestingly, our study showed that upregulating 14-3-3γ by Cap pretreatment could accumulate the markers of autophagy, however, the level of autophagy was significantly reduced when AMPK was inhibited by cotreatment with Compound C (Figures 3A–D). These results indicated that the effect of Cap in activating autophagy might be mediated via the AMPK pathway in cardiomyocytes following LPS challenge. AMPK-caused induction of autophagy may be partially related to mTOR and ULK1 (Ghavami et al., 2014). In detail, AMPK could promote 14-3-3 protein binding to Raptor (an effector of mTOR), which is needed for inhibiting mTOR activity (Meijer and Codogno, 2007). Moreover, evidence showed that the 14-3-3 protein was recruited to interfere with ULK1-mTOR binding by activating AMPK (Gwinn et al., 2008; Lee et al., 2010). Our results showed that AMPK and ULK1 were activated, but mTOR activation was suppressed in the pretreatment with the Cap group after LPS-induced toxicity. Conversely, these beneficial effects of Cap-pretreatment were offset by adding Compound C (Figures 3E–H). These data suggested that the potential mechanism of 14-3-3γ action on AMPK involves suppression of mTOR and activation of ULK1. Thus, these consistent results indicated a positive feedback during the autophagy process in LPS-damaged cardiomyocytes by Cap pretreatment.

LPS is a known stimulator in the systemic inflammatory mechanism of sepsis (Fu et al., 2015), LPS-triggered ROS generation, and the release of inflammatory cytokines, such as IL-1β, TNF-α, IL-6, and IL-10 (Ceni et al., 2014). Some studies have confirmed that 14-3-3 proteins could regulate the inflammatory response at the genetic, molecular, and cellular levels (Munier et al., 2021; McGowan et al., 2020). Cap could also inhibit inflammatory process though TRPV1-dependent or TRPV1-independent mechanisms (Fernandes et al., 2016; Braga Ferreira, et al., 2020; Ilie et al., 2019). Here, cytokine release was significantly increased in cardiomyocytes after LPS stimulation, while the high levels of IL-1β, TNF-α, IL-6, and IL-10 in the LPS group were reversed by Cap pretreatment; however, these positive changes were nullified after adding pAD/14-3-3γ-shRNA (Figure 4). Hence, our results suggested that pretreatment with Cap could alleviate inflammation by upregulating 14-3-3γ in LPS-stimulated cardiomyocytes. Moreover, autophagy plays a key role in controlling inflammation and maintaining cardiomyocyte homeostasis (Turdi et al., 2012; Zhou et al., 2018). Our findings were consistent with the above alternations of autophagy, likely because temperate activation of autophagy could scavenge damaged organelles of cardiomyocytes, which might be related to the generation of inflammatory signals. Certainly, the specific mechanism needs to be explored further.

Sepsis-induced cardiac inflammation is regulated via ROS-dependent activation (Chen et al., 2015). Our previous studies have proven that excessive ROS generation was responsible for doxorubicin-induced endotheliotoxicity and cardiotoxicity (He et al., 2020; Qiao et al., 2020). In this study, we explored the role of intracellular ROS in the LPS-stimulated cardiomyocytes. We found that intracellular ROS generation was enhanced in the LPS group (Figure 5A). Moreover, DHE, a common fluorescent probe was used to detect oxygen radicals in cardiovascular systems (Griendling et al., 2016): strong fluorescent dots were observed in the LPS group (Figures 5C,D). These changes coincided with the above results. Additionally, the endogenous antioxidant enzyme system including GSH-Px, SOD, catalase, and MDA, is the mechanism of defense against internal oxidative stress (Prasai et al., 2018). Our results showed that GSH-Px, SOD, and catalase activities were inhibited, and MDA content was increased in the cardiomyocytes after LPS injury (Figures 6A–D). The GSH/GSSG ratio maintains the redox equilibrium in cardiomyocytes by decreasing excessive ROS production (Quintana-Cabrera et al., 2012; Giustarini et al., 2016). The GSH content and GSH/GSSG ratio were reduced, while the GSSG content was significantly increased by LPS-induced cardiomyocyte injury (Figure 6E). Cap-pretreatment besides reducing intracellular ROS concentrations, also improved the activities of the endogenous antioxidant system and the abilities of the nonenzymatic antioxidant system in the cardiomyocytes after LPS-induced injury, and its protective effects were inextricably linked to the expression of 14-3-3γ (Figures 5, 6).

Mitochondria are the sites where molecules that impact the inflammation, especially the overwhelming mitochondrial ROS (mtROS), are generated (Zorov et al., 2014). We showed that mtROS generation was stimulated in LPS-treated cardiomyocytes. As expected, the curve of mtROS was significantly skewed to the left in the Cap pretreatment group (Figures 5B,C,E). Combined with prior results, we confirmed that pretreatment with Cap could protect cardiomyocytes from LPS-induced inflammation by reducing the degree of intracellular/mitochondrial ROS. Moreover, LPS triggered excessive mtROS generation, resulting in severe mitochondrial dysfunction, an overflow of the mtDNA fragment (Zhang et al., 2010) and mtROS (Brealey et al., 2002), ATP loss (Schwiebert and Zsembery, 2003) and so on. Complex Ⅰ/Ⅲ on mitochondria are the major sites of ROS generation. In our study, NDUFB8 (a subunit of mt complex I) and UQCRC2 (a subunit of mt complex Ⅲ) expression were inhibited by LPS-induced toxicity, but Cap pretreatment could promote NDUFB8 and UQCRC2 expression by upregulating 14-3-3γ levels (Figures 7A–C). These findings indicated that LPS stimulated intracellular/mitochondrial ROS by inhibiting Complex Ⅰ/Ⅲ activities in the cardiomyocytes, but these negative effects could be weakened by Cap pretreatment. Mitochondria are also essential organelles in modulating energy generation (Wang et al., 2018). The decline in ATP production is closely relevant to the impairment of mitochondrial respiration in cardiomyocytes (Makrecka-Kuka et al., 2019). Our results showed a decrease in ATP levels in the LPS-treated group, but counter outcomes were presented in the Cap pretreatment group (Figure 7D). These series of results demonstrated that pretreatment with Cap alleviated mitochondrial damage in LPS-challenged cardiomyocytes by maintaining mitochondrial metabolism, and subsequently reinforced the key role of mitochondrial function as an essential component against LPS-induced cardiotoxicity.

Mitochondrial dysfunction is sensed by a decline in MMP (De Biasi et al., 2015); simultaneously, damaged mitochondria release mitophagy-related factors such as Parkin and BNIP3 to modulate the mitophagy pathway (Sun et al., 2018) and promote the opening of mPTPs (Hamacher-Brady et al., 2007; Zhang et al., 2008). In LPS-induced cardiomyocyte injury, pretreatment with Cap could sustain MMP and inhibit mPTP opening, while the downregulation of 14-3-3γ presented an opposite trend (Figures 7E,F). These results suggested that mitochondria were the major organelles of LPS-induced cardiotoxicity, and might also be the targets for Cap in protecting cardiomyocytes against LPS-induced injury via 14-3-3γ expression upregulation.

## Limitation of the Study

Mitophagy significantly influences LPS-induced cardiotoxicity (Sun et al., 2018). Further studies are needed to explore the potential mechanism of Cap-upregulated 14-3-3γ expression, and how the AMPK-mTOR/ULK1 pathway regulates IL-1β, TNF-α, IL-6, and IL-10 in LPS-stimulated cardiotoxicity during the mitophagy process.

## Conclusions

**FIGURE 8:**
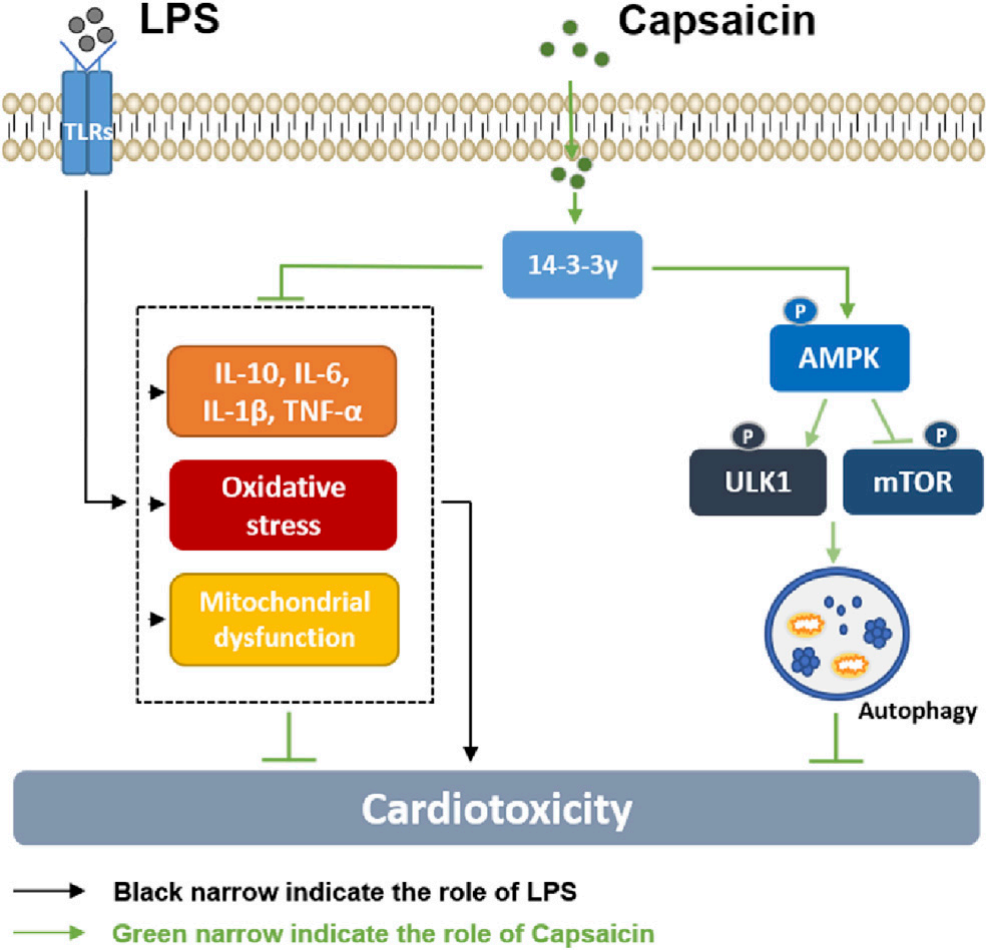
Diagram showing the possible mechanism by which Cap alleviates LPS-induced cardiotoxicity. LPS-induced cardiotoxicity was manifested in activating inflammatory cytokines, producing excess oxygen radicals, and causing mitochondrial damage. The negative effects of LPS on cardiomyocytes are reversed by Cap, and the underlying mechanism may be involved in Cap-mediated 14-3-3γ expression and autophagy regulation via the AMPK-mTOR/ULK1 pathway.

In this study, we investigated the possible mechanism of Cap-upregulated 14-3-3γ expression in cardioprotection against LPS-induced injury. LPS-induced cardiotoxicity was manifested in activating inflammatory cytokines, producing excess oxygen radicals, and triggering mitochondrial damage. However, the negative effects of LPS on cardiomyocytes are reversed by Cap, and the underlying mechanism may be involved in Cap-mediated 14-3-3γ expression and autophagy regulation via the AMPK-mTOR/ULK1 pathway (Figure 8).

## Data Availability

The raw data supporting the conclusions of this article will be made available by the authors, without undue reservation, to any qualified researcher.

## Abbreviations

AMPK, AMP-activated protein kinase; BafA, Bafilomycin A1; Cap, Capsaicin; CK, creatine phosphate kinase; DHE, dihydroethidium; LDH, lactate dehydrogenase; LPS, lipopolysaccharide; MDC, dansylcadaverine; mTOR, mammalian targets of rapamycin; MMP, mitochondrial membrane potential; mPTP, mitochondrial permeability transition pore; mtROS, mitochondrial ROS; NDUFB8, NADH dehydrogenase [ubiquinone] 1 beta subcomplex subunit 8; NRCMs, neonatal rat cardiomyocytes; NS, nonsignificant; ROS, reactive oxygen species; ULK1, Unc-51 like- autophagy-activating kinase 1; UQCRC2, cytochrome b-c1 complex subunit 2.
